# Heparin and Related Drugs: Beyond Anticoagulant Activity

**DOI:** 10.1155/2013/910743

**Published:** 2013-07-30

**Authors:** Clive Page

**Affiliations:** Sackler Institute of Pulmonary Pharmacology, Institute of Pharmaceutical Science, King's College London, 5th Floor, Franklin-Wilkins Building, Waterloo Campus, 150 Stamford Street, London SE1 9NH, UK

## Abstract

Heparin has been widely used as an anticoagulant for more than 80 years. However, there is now considerable evidence that heparin also possesses anti-inflammatory activity, both experimentally and clinically. Importantly in many instances, the anti-inflammatory actions of heparin are independent of anticoagulant activity raising the possibility of developing novel drugs based on heparin that retain the anti-inflammatory activity. Heparin exhibits anti-inflammatory activities via a variety of mechanisms including neutralization of cationic mediators, inhibition of adhesion molecules, and the inhibition of heparanase, all involved in leukocyte recruitment into tissues. It is anticipated that furthering our understanding of the anti-inflammatory actions of heparin will lead to the development of novel anti-inflammatory drugs for a variety of clinical indications.

## 1. Introduction

Heparin has been used for over eighty years as an anticoagulant. Despite its widespread use, the exact mechanism for the anticoagulant activity of heparin was not elucidated until the 1960s and the specific polysaccharide sequence within the heparin molecule required for this interaction was not defined until nearly twenty years later [1]. The inherent nature of heparin being a polydisperse heterogeneous molecule continues to make this a complex material to work with. In addition to the well described anticoagulant effect of heparin, a range of polysaccharides, some derived from heparin, and some from related structures, have been found to interact with a wide variety of biological pathways and systems, raising the possibility that such drugs may have wider therapeutic uses than inhibiting coagulation. These other activities of heparin and related drugs are less well understood than anticoagulant activity, but such drugs are now under investigation for a wide range of clinical indications, particularly for the treatment of inflammatory diseases.

**Figure 1:**
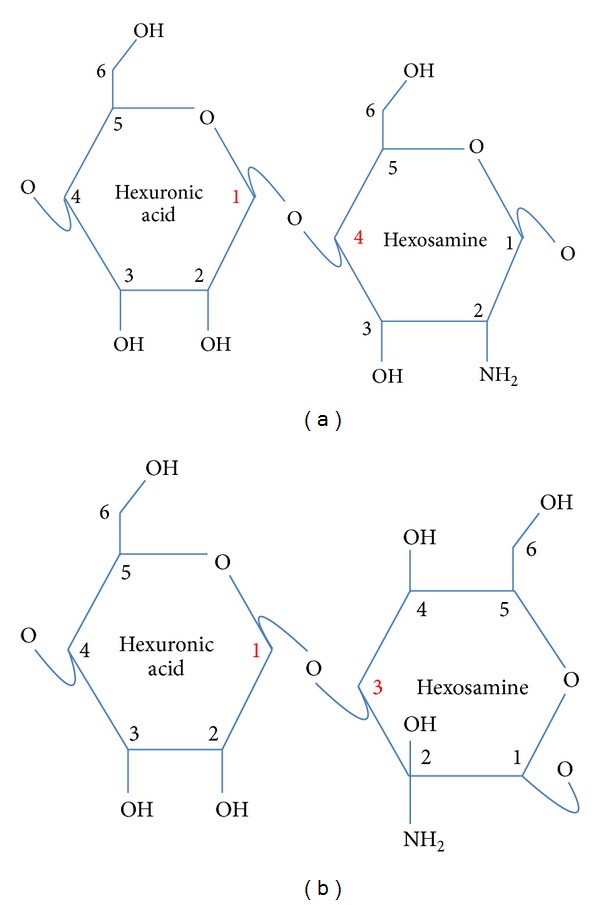
Generic disaccharide backbone structure of glycosaminoglycans with (a) 1–4 linkage, common to heparin and heparan sulphate and (b) 1–3 linkage, common to chondroitin and dermatan sulphates. Numbers on the rings relate to the carbon position, hydroxyl, and amine groups can be sulphated and alternatively orientated.

Heparin is a polysaccharide, and heparin has several unusual characteristics. Firstly, it is polydisperse in nature; that is, it does not possess a defined single structure in the manner of a simple low-molecular-weight drug such as aspirin. Rather, heparin contains a range of saccharide chains of variable lengths and structural diversity and will typically have an average molecular weight of 14 to 18 kDa, but can contain polysaccharides from 10 to over 100 monosaccharide units [1]. The second feature is that heparin is a highly sulphated molecule, and due to this property has a very high negative charge which allows it to bind to a very wide array of positively charged biological materials (see [2] for a review). Heparin belongs to the glycosaminoglycan (GAG) family of polysaccharides which are characterised by alternating hexuronic acid and hexosamine disaccharides as the backbone structure (see Figure 1), although there are a number of other molecules that fall into the GAG family (see Table 1).

Unfractioned heparin and low-molecular-weight heparins (formed from fractionation or degradation of heparin by different chemical methods, e.g., tinzaparin—(by enzymatic digestion, dalteparin—by nitrous acid depolymerisation, enoxaparin—alkaline *β*-elimination, and parnaparin—oxidative depolymerisation)) are widely used as anticoagulants in a range of different clinical indications. However, in mammals, heparin is uniquely found in mast cells, which reside within mucosal and connective tissues suggesting that physiologically heparin may be involved in the regulation of inflammatory responses. Indeed, I suggested in 1991 that endogenous heparin may well serve as an endogenous regulator of the inflammatory response in much the same way as various mechanisms have been shown to homeostatically regulate the actions of neurotransmitters and hormones [3]. Mast cells contain an array of inflammatory mediators packed in to their granules which are released on stimulation, and heparin has been found packed in conjunction with a range of cationic molecules, for example, chymase and tryptase. Due to the high negative charge of heparin, a widely held view has been that heparin serves as a packing agent, allowing the containment and storage of large quantities of these various positivity charged mediators in very close proximity [4]. However, it is now recognized that many different proteins involved in the inflammatory cascade have heparin-binding domains in their structure allowing them to recognise and bind heparin, and in many cases heparin is able to inhibit the action of these proteins (see [2]). Thus, endogenous heparin may have an important role in helping control the localised inflammatory response, rather than the coagulation system and indeed heparin has been shown to be released by mast cells [5, 6] and circulating heparin like material has been identified in patients with allergy [7], presumably due to the repeated mast cell degranulation that occurs in such patients.

In addition to endogenous heparin being an anti-inflammatory agent, there are now many experimental and clinical studies demonstrating positive anti-inflammatory activities of heparin (see below), suggesting that such activities could be exploited for therapeutic use. However, the use of heparin itself as an anti-inflammatory drug is currently limited by the anticoagulant activity of the molecule, notwithstanding the observations that when heparin is applied by alternative topical routes of administration, for example, inhalation, and even at higher than conventional anticoagulant doses, no significant anticoagulant effects have been noted systemically [8, 9]. However, the observations, by a number of laboratories, that the anti-inflammatory actions of heparin are independent of its anticoagulant activity [10–13] have spurred the field into investigating novel molecules that mimic the anti-inflammatory activities of heparin, whilst lacking anticoagulant activity and these will be discussed in further detail below.

## 2. Nonanticoagulant Effects of Heparin

### 2.1. Effects on Inflammatory Mediators

Heparin can inhibit the activation of a range of inflammatory cells [14–25], an effect that is due in part to the binding and neutralisation of inflammatory mediators and enzymes released during an inflammatory response (reviewed by [2]) that would otherwise go on to activate such cells. Likewise, certain enzymes and cytotoxic mediators released from these cells, involved in propagation of the inflammatory response and subsequent tissue damage and remodelling, have also been shown to be inhibited by heparin, including elastase [26, 27], cathepsin G [26], eosinophil peroxidase [28], eosinophil cationic protein [29], major basic protein [30], certain cytokines (reviewed by [31]), and chemokines (reviewed in [32]).

Many growth factors, including basic fibroblast growth factor [33] and transforming growth factor-beta [34, 35], both of which are involved in the regulation of smooth muscle proliferation, (a feature of the tissue remodelling seen in diseases including asthma, atherosclerosis, and coronary stenosis), are bound by heparin. A long established property of heparin is that of inhibition of vascular smooth muscle cell proliferation [36], an effect which is known to be independent of the anticoagulant actions of heparin [6], and which extends to airway smooth muscle [35, 37, 38]. 

Heparin is also known to inhibit the degranulation of isolated human mast cells in response to a variety of stimuli, and hence inhibit the release of histamine [20, 39]. This effect is considered to be due to inhibition of inositol 1,4,5-triphosphate- (IP3-) dependent calcium release by heparin. The action of IP3 on the endoplasmic reticulum is potently and competitively blocked by heparin applied to permeabilised mast cells in vitro [40]. IgE-mediated degranulation of mast cells in vitro was found to be inhibited by two fractions of heparin, one which lacked anticoagulant activity and was actually the more potent preparation in this respect, evidence that this effect also does not depend upon the anticoagulant effects of heparin [16]. The cytotoxic effects of TNF-*α*-activated eosinophils on endothelial cells are also markedly inhibited by heparin [24], as is the homotypic aggregation and chemotaxis of eosinophils in response to complement factor C5a, another inflammatory mediator bound by heparin [25, 41]. Furthermore, unfractionated heparin inhibits lipopolysaccharide-induced activation of endothelial cells via inhibition of p38 MAPK and NF-KB [42].

Heparin has been shown to bind to the surface of neutrophils [43] and can inhibit their degranulation [18, 21], homotypic aggregation [17, 18, 44, 45], the production of superoxide anions, the activity of lysosomal enzymes [17], and the ability of neutrophils to activate platelets [17, 19], again in a manner that is not dependent upon anticoagulant activity. Furthermore, heparin is able to inhibit neutrophil activation in response to thrombin-stimulated platelet products, in addition to inhibiting thrombin-induced platelet aggregation [22], and at high concentrations, platelet *α*-granule secretion is inhibited [23].

### 2.2. Effects on Cellular Adhesion

An important component of the inflammatory response is the adherence of inflammatory cells to the vascular endothelium and their subsequent diapedesis into tissues. This is now a well-characterized process, and heparin has been shown to inhibit each of the different stages involved in inflammatory cell recruitment into tissues (reviewed in [2]). Thus, heparin has been shown to inhibit leucocyte-endothelial adhesion, both in vitro (reviewed in [2, 17, 46, 47]) and in vivo [13, 48–53], as well as to limit the ultimate accumulation of cells in inflamed tissues, in response to both allergic [11, 54–56] and nonallergic [13, 48, 50, 57, 58] stimuli. 

Heparin is known to bind directly to several adhesion molecules expressed during inflammation and the structural requirements for these interactions are becoming increasingly well characterised (e.g., reviewed by [59]). On leucocytes, L-selectin, a molecule involved in early adhesive interactions between inflammatory cells and the vessel wall, is bound by heparin [60], and endothelial heparan sulphate is able to act as an endothelial ligand for this molecule during cell rolling [61]. The *β*2-integrin adhesion molecule mac-1 (macrophage-1; CD11b/CD18), important for the firm adhesion of leucocytes to endothelium, is also bound by heparin [62, 63] to an extent that surface immobilised heparin is able to support mac-1-dependent neutrophil adhesion under flow conditions in vitro [62]. Therefore, soluble heparin may inhibit mac-1-dependent interactions between leucocytes and the endothelium; the effects of heparin on leucocyte adhesion in vivo have been found to be dependent on such an interaction with mac-1 [51]. On endothelial cells, heparin binds to P-selectin [64], a selectin adhesion molecule involved in the early sequestration of neutrophils during inflammation. Indeed, the antimetastatic effects of heparin can be ascribed, at least in part, to inhibition of P- and L-selectin function [65, 66]. The selectins are a family of glycoprotein adhesion molecules comprising an epidermal growth factor (EGF) like moiety, repeating sequences mimicking those found on complement binding proteins and an NH2-terminal lectin domain. It is via the lectin domain that these molecules Ca^2+^-dependently bind to carbohydrate structures on the surfaces of interacting cells. Selectins are concerned predominantly with the rolling stages of adhesion, without which firm adhesion and transmigration cannot proceed [67]. However, despite structural congruencies between the selectins, it has been demonstrated that heparin is unable to bind to E-selectin [60]. This difference is known to rely upon two specific amino-acid residues in the EGF-like domain of the selectins, in that if these residues are altered, E-selectin can be made to bind heparin, and the ability of P-selectin to bind heparin diminished [68]. This differential effect may possess physiological significance with respect to the role of endogenous heparin and, possibly, heparan sulphate in the inflammatory process. Indeed, a similar selectivity of binding can be observed amongst key members of the immunoglobulin superfamily adhesion proteins. Heparin has been shown to bind PECAM-1 (platelet endothelial cell adhesion molecule-1 [69]), an IgSF-adhesion molecule thought to be involved in leucocyte transmigration due to its location at intercellular junctions on the endothelium. The homotypic aggregation of PECAM-1-transfected fibroblasts was found to be inhibited by heparin in a manner dependent upon interaction with the second immunoglobulin domain [70]. Similarly, heparin is able to bind directly to neuronal cell adhesion molecule (NCAM), through a heparin-binding region located on the second immunoglobulin domain [71], as well as through a further heparin-binding region on the first immunoglobulin domain [72]; such interactions with heparan sulphate are important for the physiological functioning of this protein in neuronal development [73]. However, the IgSF-adhesion molecules intercellular adhesion molecule- (ICAM-) 1 and ICAM-2, expressed on vascular endothelium and ligands for leucocyte *β*2-integrins, do not appear to be bound by heparin. However, given that heparin can affect the functioning of ICAM-1 indirectly, by binding of mac-1, it is plausible that the cell trafficking associated with physiological immune surveillance, facilitated by, for example, interactions between lymphocyte function-related antigen (LFA-1; CD11a/CD18) and ICAM-1/2 may be spared while those associated with excessive cell recruitment during inflammation may be inhibited.

### 2.3. Inhibition of Heparanase

The ubiquitous distribution of heparan sulphate proteoglycans (HSPGs) in mammalian systems provides a clear indication of the physiological importance of these molecules, which are thought to contribute to growth and development, are key structural components of extracellular matrices, and are involved in the localisation and bioactivity of a wide array of mediators, including enzymes, growth factors, cytokines, and chemokines (reviewed by [74, 75]). The endo-*β* glucuronidase heparanase (HPSE1) is responsible for the site-selective cleavage of heparan sulphate chains, thus regulating the activity of the wide range of proteins that is functionally dependent upon HSPG. HPSE1 has now been sequenced and cloned [76–79] and HPSE1 exists as a 50 kDa and 8 kDa heterodimer processed from a single, inactive 65 kDa proenzyme ([76] reviewed by [80]). The catalytic sites on the enzyme have also now been characterized [81]. 

HPSE1 activity has been demonstrated in spleen, lymph nodes, leucocytes and platelets, as well as in endothelial and smooth muscle cells. Moreover, the well-accepted role of HPSE1 in cancer (reviewed by [82]) is underscored by the fact that in human tumours, mRNA for HPSE1 is markedly increased with respect to corresponding normal tissues and that HPSE1 activity in tumour cells has been found to correlate positively with metastatic potential [83]. There are many similarities between leucocyte diapedesis and tumour cell metastasis, and given the evidence that in HPSE1 is involved the latter, it is perhaps not surprising that this enzyme has also been reported as a potential target for novel anti-inflammatory drugs [82].

The potential importance of heparanase activity is illustrated by the fact that in tissue sections from inflammatory bowel disease patients, when compared to healthy tissues, areas of extensive GAG disruption are visible on vascular endothelium and basement membrane, which correlate with localised areas of inflammation [84] and increased levels of GAG degradation products have been found in the urine of patients with asthma, which is thought to reflect the breakdown of extracellular matrices as a result of the inflammatory processes in the airway [85].

It is, therefore, of interest that heparin has long been known to be an inhibitor of HPSE1 activity [86], and it is also well established that heparan-degrading enzymes are released by certain leucocytes during the process of diapedesis [87, 88]. Indeed, when heparin is used at low doses in lymphocyte-driven inflammatory processes such as allergic encephalomyelitis [89, 90], delayed-type hypersensitivity (DTH) [10] and graft-versus-host reactions [91, 92], leucocyte infiltration into tissues is markedly inhibited, and it has been suggested that this effect is via inhibition by heparin of HPSE1. However, recent data with selective inhibitors of HPSE1 have not confirmed anti-inflammatory activity with such drugs in nonallergic inflammatory models, perhaps questioning a central role for this enzyme in leucocyte infiltration (D. Spina, Personal Communication). It has further been demonstrated that vascular endothelial cells also secrete heparanase and that exposure of endothelial cells to proinflammatory cytokines upregulates this secretion [93, 94], further suggesting an important role for this enzyme in inflammation. Moreover, the development of DTH reactions has been found to correlate with endothelial heparanase expression in mice [94].

### 2.4. Effects on Acute Inflammatory Responses

In animal studies, pretreatment with heparin has been shown to inhibit eosinophil infiltration into the inflamed lung [54, 55, 95] and skin [57], neutrophil accumulation in the inflamed peritoneal cavity [13, 50], independently of anticoagulant activity [11, 13], and to inhibit vascular permeability induced by certain autacoids [96, 97] or the bacterial formyl peptide [97]. Additionally, platelet-activating factor-induced bronchial hyperresponsiveness was inhibited by heparin administration in rabbits [54] and similar effects have been reported in an allergic sheep model, whereby inhaled heparin was found to inhibit the acute airway responses to inhaled allergen [15], an effect that was shared by very low-molecular-weight and nonanticoagulant heparins [16], and in guinea pigs, whereby the protective effect of heparin against bronchial hyperresponsiveness to methacholine was suggested to be due to preservation of nitric oxide signalling in the airway [98].

Heparin has been found, in a number of preclinical models, to protect against ischaemia-reperfusion injury. Thus, in a hamster dorsal skin chamber model, leucocyte-endothelial adhesion induced by ischaemia-reperfusion is inhibited by heparin pretreatment [99], as is cardiac muscle damage [100]. Furthermore, administration of heparin subsequent to transient focal cerebral ischaemia in rats was found to reduce the degree of brain injury by inhibiting reperfusion-induced leucocyte accumulation [58]. Heparin has also recently been suggested as a plausible agent for limitation of the delayed neurological injury that follows subarachnoid haemorrhage [101], by virtue of its broad anti-inflammatory effects. Clearly, the potential to promote further haemorrhage in this setting is a legitimate concern, but it has been suggested that systemic, subanticoagulant doses of heparin may be sufficient to elicit beneficial effects in this condition [101], and, moreover, intracisternal administration of heparin has been found to be protective following experimental subarachnoid haemorrhage in rats [102]. However, given that the anti-inflammatory properties of heparin appear largely to be separable from its effects on coagulation, nonanticoagulant heparin-like molecules may provide a safer approach to treating this important clinical problem in the future. 

It has long been appreciated that the anti-inflammatory effects of heparin observed preclinically have also been extended into a number of clinical settings. Heparin has potential use in human inflammatory disease and was first assessed for this purpose in the 1960s, in small, subjectively assessed trials [103, 104]. More recently, in controlled studies, heparin has shown potential in the management of clinical asthma [8, 14, 105, 106] and chronic obstructive pulmonary disease (COPD) [107, 108]. In patients with allergic rhinitis, topical heparin has been observed to reduce eosinophil recruitment into the nose [56] following allergen exposure and to be of value in the treatment of inflammatory bowel disease ([109–111]; reviewed by [112]), although meta-analyses of these trials have concluded that there is currently insufficient evidence to support the use of heparin for the treatment of active ulcerative colitis [113, 114]. 

Importantly, however, in none of these clinical studies was heparin treatment found to elicit significant haemorrhagic side effects, either when administered systemically or locally. Indeed, in a study performed specifically to address the effects of inhaled heparin on coagulation parameters [9], it was found that almost 40% of a single inhaled dose of heparin is detectable in the lung 24 h later, with no significant effects on blood coagulation. However, given that the anticoagulant actions of heparin appear not to be necessary for the majority of beneficial effects seen in models of inflammation, it seems likely that novel drugs which retain the anti-inflammatory effects of the parent heparin molecule, without the anticoagulant effects will be useful in the management of inflammatory diseases that have been found to respond positively to the administration of heparin or low-molecular-weight heparin; for example, selectively 2,3-O-desulphated heparin, which is currently in clinical trials for COPD, is one such approach [12]. 

## 3. Effects of Heparin in Cancer

Due to the common use of heparins for the prophylaxis of venous thromboembolism (VTE) in cancer patients, a sizeable body of evidence exists to suggest that heparin confers benefit in the treatment of cancer that is additional to the direct effects of this drug on blood coagulation (see [115–122]). Analysis of trials of heparin treatment in cancer patients indicates an improved rate of survival [117] and meta-analyses performed specifically to assess the effects of heparin and LMW heparin treatment on survival in cancer patients have indicated positive effects [123, 124]. 

As discussed previously, the accumulation of metastatic tumour cells into tissues, like leucocytes, is dependent upon adhesion to the vascular endothelium and subsequent diapedesis and many similarities exist between the processes utilised by inflammatory cells and tumour cells in this respect (reviewed by [125]), including a dependency on platelet activation [126, 127]. However, the involvement of anticoagulant mechanisms in these effects is less clear than is perhaps the case for many of the anti-inflammatory properties of heparin. Heparin has been demonstrated repeatedly to reduce metastasis of carcinoma cells in animal models (e.g., [80, 128–131]). However, with respect to the contribution of the anticoagulant effects of the drug, it has been suggested that the basis of the antimetastatic effects of unfractionated heparin lies in the inhibition of fibrin deposition around tumour cells, a factor considered to protect the cells from immune attack [128]. Nonetheless, many studies have found that fractions of heparin with much reduced anticoagulant activity, or none at all, also inhibit metastasis (e.g., [129, 131]). Specific mechanisms thought to be involved in this effect include inhibition of heparanase activity [116], selectin function [126] and the tissue factor pathway [132]. Tissue factor can promote angiogenesis and metastasis via mechanisms both related and unrelated to plasma coagulation [118]. It has been suggested that the effects of heparin and related molecules in models of tumour growth and metastasis rely, at least in part, on the promotion of tissue factor pathway inhibitor (TFPI) release from endothelial cells [132]. Regarding selectin function, clinically relevant levels of LMW heparin, with respect to anticoagulation, have been shown to inhibit experimental metastasis in a manner that correlates with the ability to inhibit P- and L-selectin function; the pentasaccharide fondaparinux, which lacks this ability, was found to be without effect in the same assays, at levels normalised for anticoagulant activity [65], suggesting that it is not the anticoagulant effects per se of heparin that contribute most significantly to effects on tumour cell metastasis. Moreover, mice deficient in both P- and L-selectin were found to be protected against experimental metastasis and, importantly, in these mice, treatment with heparin conferred no further protection [66], in contrast to the marked effects seen in wild-type animals in a range of studies. Protective effects of heparin in cancer models extend beyond the inhibition of metastasis to include those on tumour growth and angiogenesis. Heparin has long been known to be antiangiogenic and its inhibitory effects on heparanase are again well established to be involved in this effect (reviewed by [133]). Growth-factor-induced endothelial cell proliferation is inhibited by unfractionated and LMW heparins [134–136]. Whilst standard LMW heparins in this respect were found to be more potent than unfractionated heparin [135, 136], ultralow-molecular-weight species, including the anticoagulant pentasaccharide fondaparinux, were without effect [136]. Moreover, antiangiogenic and antimetastatic effects may further be mediated through interference with the chemokine system, which is known to be involved in these phenomena (reviewed by [137]).

Therefore, it is likely that heparins inhibit angiogenesis and metastasis via an array of mechanisms, including but by no means limited to heparanase inhibition.

## 4. Effects of Heparin on Wound Healing and Tissue Repair

Administration of heparin by inhalation has been found to be a viable option for the management of smoke inhalation injury in survivors of fire (reviewed by [138, 139]), which reduces the acute lung injury that contributes significantly to morbidity and mortality in these patients, both alone and in combination with *N*-acetylcysteine [140].

Indeed, there are a number of reports of heparin being used, topically or systemically, to treat burns, although there are a lack of effectively controlled studies in this area for clear conclusions to be drawn as to the efficacy of this approach (reviewed by [141]). However, isolated case reports continue to emerge, suggesting that heparin is able to promote tissue repair and inhibit inflammation in burns patients (e.g., [142]). Whilst controlled, randomised studies are required to assess the potential utility of heparin in this setting, the demonstrated anti-inflammatory effects in other experimental systems do indicate the potential for a useful effect. Furthermore, in animal models, application of heparin-binding epidermal growth factor-like growth factor (HB-EGF), which is known to be upregulated both in human burn tissue and during healing of experimental burn tissue [143], has been shown to promote healing of partial-thickness burn injuries specifically through potentiation of the expression of transforming growth factor-*α*, another member of the EGF family of growth factors involved in wound repair [144], and to promote healing of ileal tissue following experimental reanastomosis surgery [145]. It has recently been reported that tissue localisation of HB-EGF, through binding to HSPG, mediates the function of the growth factor as a juxtacrine inhibitor of cell proliferation and that disruption of this binding allows the released HB-EGF to function as an autocrine mitogen [146]. Therefore, it is possible that soluble heparin at the site of injury could act competitively to release this growth factor from heparin sulphate binding sites and promote its participation in tissue repair. Moreover, topically applied heparin has been found to promote effective tissue repair in rabbit trachea, in a model of tissue healing following airway surgery [147], further suggesting that the immunomodulatory effects of heparin may be useful in the specific situation of tissue repair following localised injury. 

## 5. Other Conditions Benefiting from Heparin Treatment

Heparin [148] and the related molecule, pentosan polysulphate, have been shown to have beneficial activity in the treatment of interstitial cystitis and indeed the latter drug has been approved for such use in a number of countries. Another interesting area is the potential use of heparin(s) to treat and prevent protracted labour. Some clinicians had noted that when pregnant women were administered low-molecular-weight heparins (LMWHs) for the prevention of thrombosis, there was a shorter induction time to labour [149–151]. It has been suggested that this effect may relate to inhibition of IL-8. Recent phase 2 clinical studies conducted with tafoxiparin sponsored by Dilafor have confirmed that this LMWH is effective in reducing the incidence of extended labour (http://www.dilafor.com/).

Another possible use of heparin in the treatment of cystic fibrosis was presented [152] and early clinical studies have reported that heparin administered by inhalation provides clinical benefit in patients with COPD (http://www.vectura.com/) which may result from the ability of heparin to act as a mucolytic agent [153] and/or via its well documented effect on neutrophil activations (see above), a major inflammatory cell infiltrating the lung of patients with cystic fibrosis. 

## 6. New Approaches to Treatment

There has long been interest in developing heparin-based anticoagulants that do not require parenteral administration and with respect to, for example, the management of chronic inflammatory diseases, the need for convenient and acceptable methods of drug delivery is arguably an even greater issue. In some circumstances, such as inflammatory diseases of the lung, local administration of heparin by inhalation is an option, but where systemic effects are required, an efficient and predictable drug absorption profile becomes necessary. Absorption of unmodified, unfractionated, and LMW heparins has been reported following oral administration in rats [154–156], and in rats and humans when administered with the absorption-enhancing delivery agent sodium N-[8(2-hydroxybenzoyl)amino]caprylate (SNAC) [157–160]. Similarly, augmentation of heparin absorption via the pulmonary [161–167] and nasal [168–170] routes has been described, when the drug is coadministered with delivery systems including polyethyleneimines, cyclodextrins, alkylmaltosides, alkanoylsucroses, poly-L-arginine, and within PEGylated nanocarriers. Moreover, heparin has been administered successfully in validation studies of needle-free injection devices [171–173], designed to reduce the pain and inconvenience associated with the regular administration of substances such as insulin, presenting a possible alternative to conventional subcutaneous injection of heparins. In all of these studies, measurement of coagulation parameters was used to assess the efficacy of heparin delivery. However, the fact that a robust and well-characterised effect of heparin can be measured, following the administration of standard heparins by nonstandard routes, is promising with respect to the potential delivery of heparin species for nonanticoagulant uses. 

A number of new approaches are being investigated to exploit these non-anticoagulant actions of heparin. Heparin and LMWHs are being investigated in a range of diseases (see Table 2) and molecules such as the nonanticoagulant o-desulphated heparin [12]) are in clinical development (Table 2). In addition, a number of polysaccharides of different length and sulphation patterns have been described in the literature [16, 21, 174–178] which are undergoing preclinical and clinical investigation for a range of diseases.

Additionally, a number of GAG analogues that bind cytokines are under development as novel anti-inflammatory drugs [48, 179]. There is also considerable interest in novel polysaccharides from marine sources such as fucoidans, as anti-inflammatory drugs (see [180]).

## Figures and Tables

**Table 1:** Examples of glycosaminoglycans (GAGs) and their main backbone components.

Table of GAGs	Hexuronic acid	Hexosamine	Glycosidic linkage	Average sulphate per disaccharide
Hyaluronic acid	GlcUA	*β*GlcNAc	1–3, 1–4	0
Chondroitin sulphate	GlcUA or GlcUA(2S)	GalNAc or GalNAc(4S) or GalNAc(6S) or GalNAc(4S,6S)	1–3, 1–4	0–2
Heparin	GlcUA or IdoUA(2S)	GlcNAc or GlcNS or GlcNAc(6S) or GlcNS(6S)	1–4	2-3
Heparan sulphate	GlcUA or IdoUA or IdoUA(2S)	GlcNAc or GlcNS or GlcNAc(6S) or GlcNS(6S)	1–4	0–2
Dermatan sulphate	GlcUA or IdoUA or IdoUA(2S)	GalNAc or GalNAc(4S) or GalNAc(6S) or GalNAc(4S,6S)	1–3, 1–4	0–2

Hexuronic acid—GlcUA: *β*-D-glucuronic acid; GlcUA(2S): 2-O-sulfo-*β*-D-glucuronic acid; IdoUA(2S): 2-O-sulfo-*α*-L-iduronic acid; IdoUA: *α*-L-iduronic acid.

Hexosamine—*β*GlcNAc: *β*-D-N-acetylglucosamine; GlcNAc: *α*-D-N-acetylglucosamine; GalNAc: *β*-D-N-acetylgalactosamine; GalNAc(4S): *β*-D-N-acetylgalactosamine-4-O-sulfate; GalNAc(6S): *β*-D-N-acetylgalactosamine-6-O-sulfate; GalNAc(4S,6S): *β*-D-N-acetylgalactosamine-4-O, 6-O-sulfate; GlcNS: *α*-D-N-sulfoglucosamine; GlcNS(6S): *α*-D-N-sulfoglucosamine-6-O-sulfate.

**Table 2:** Some examples of clinical trials where heparin is used as a treatment beyond anticoagulant activity (taken from the US National Institute of Health online database http://www.clinicaltrials.gov/ and EU Clinical Trials Register http://www.clinicaltrialsregister.eu/ in Sept., 2012).

Condition	Intervention	Purpose	Phase	Trial identifier(s)
Infertility	Heparin, nadroparin	Heparin increases outcome in poor responders undergoing in vitro fertilisation	Phases 2 and 3	NCT01315093 2011-003080-30

Haemodialysis	Heparin	Evaluates if topically applied heparin to placebo on suitability of constructed primary arteriovenous fistulas	Phase 2	NCT01382888 2011-000455-16

Inhalation burns, smoke inhalation injury	Heparin	Efficacy of nebulised heparin on lung injury score in inhalation burn injury over normal care	Phase 2	NTC01454869

Lung cancer	Tinzaparin/enoxaparin	LMWH can inhibit tumour growth and metastasis and enhance survival of patients	Phase 3	NCT00475098, NCT00771563, and 2007-007696

Inflammation	Enoxaparin	Treatment of inflammation in intraocular lens implantation and chronic glomerulonephritis	Phase 4 Phase 3	NCT00986076 2005-002989-11

Adenocarcinoma of the colon	Tinzaparin	LMWH reduction of metastases and recurrence in patients as seen in animal models	Phase 3	NCT01455831

Breast, colorectal, Lung, prostate, and venoocclusive cancers	Dalteparin, nadroparin	Assesses if LMWH beyond inhibition of thrombosis improves quality of life over standard treatment	Phases 2 and 3	NCT00003674, 2005-005336-27

Vulvodynia	Enoxaparin	LMWH may reduce pain in women with vulvodynia	Phase 2	NCT00874484

Ulcerative colitis	Deligoparin	uLMWH may help to reduce inflammation in ulcerative colitis	Phases 2 and 3	NCT00033943

Diabetic foot ulcers	Dalteparin	Treatment on chronic foot ulcers due to peripheral arterial occlusive disease in diabetics	Phases 2 and 3	NCT00765063

Ovarian cancer	Dalteparin	Assesses the disease response with LMWH over standard therapy	Phase 2	NCT00239980

Metastatic Pancreatic cancer	O-Desulphated heparin (ODSH)	Determines if ODSH is efficacious in patients receiving normal therapy	Phase 2	NCT01461915

Pregnancy complications	Enoxaparin	Evaluate the efficacy of LMWH on pregnancy outcome in women with previous pregnancy complications	Phase 3	2006-004205-26

Burns	Heparin	Assess analgesic effect of heparin in topical and parenterally treatment	Phase 2	2007-004304-12

Cystic fibrosis	Heparin	Nebulised heparin on easing cystic fibrosis	Phase 2	2007-006276-11

Pulmonary conditions	Heparin, desulfated heparin	Improves lung function in obstructive pulmonary conditions	Phase 2	2006-006378-32, 2010-024168-16

Labour	Nonanticoagulant LMWH	Reducing prolonged labour	Phase 2	2006-005839-20

Microalbuminuria	Sulodexide (LMWH and dermatan sulphate)	Treats microalbuminuria in type 2 diabetes	Phase 3	2005-003158-91
